# Unusual posteromedial tubercle fracture extending to the talar dome

**DOI:** 10.1002/ccr3.3318

**Published:** 2020-09-10

**Authors:** Eleni Pappa, Michael Michalos, Konstantinos Zisis, George Koundis, Constantine Kokoroghiannis, Dimitrios Evangelopoulos

**Affiliations:** ^1^ 5th Department of Orthopaedics “KAT” General Hospital of Athens Athens Greece

**Keywords:** ankle sprain, Cedell's fracture, talar medial tubercle fracture

## Abstract

The present case is unique in that the fracture of the posteromedial talar tubercule involved the tibiotalar rather than the subtalar joint as described in existing reports, which underlines the need for acute management by the Orthopaedic Surgeon

## Graphical abstract

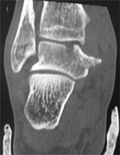

## 1 CASE REPORT

**FIGURE 1:**
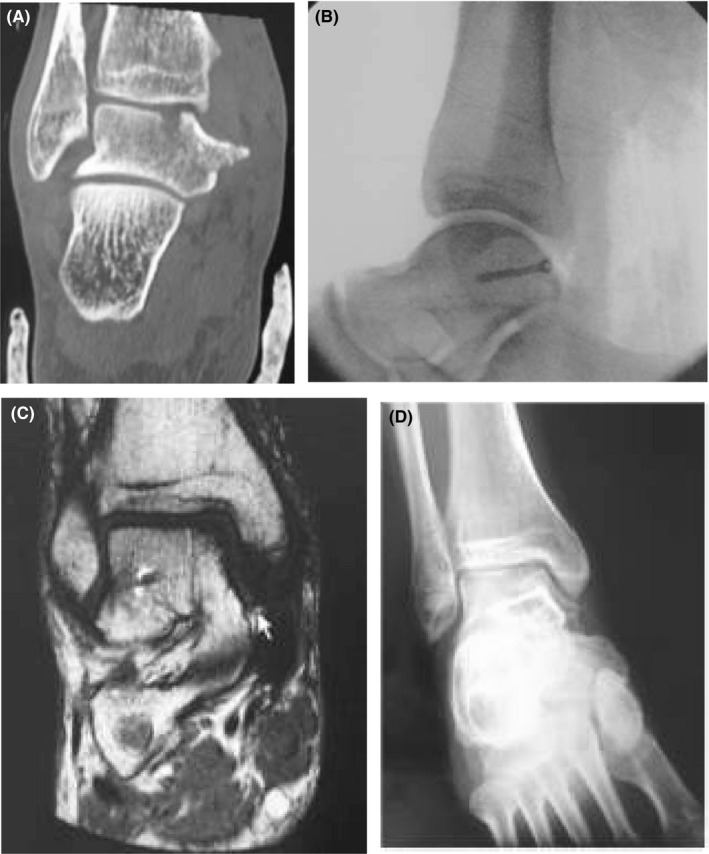
A, Vertical slice of the initial computed tomography scan showing the fracture of the posteromedial tubercle and the posteromedial talar dome which extends to the tibiotalar joint. B, Intraoperative radiograph of the open reduction and the internal fixation of the fracture with a 3.5 mm cancellous screw. C, MRI examination during the last follow‐up, 3 y postoperatively, showing the undisplaced cancellous screw in the talar body, in both slices. D, Radiograph during the last follow‐up, 3 y postoperatively, showing the undisplaced cancellous screw in the talar body, as well as the absence of post‐traumatic arthritis of the tibiotalar joint

Fractures involving the medial tubercle of the posterior process of the talus involving the tibiotalar joint are extremely rare and usually confused with ankle sprains that are very common.^1^, ^2^ A 45‐year‐old man presented at the emergency department complaining of acute pain of the right ankle and inability to bear weight after entrapping his right foot in a ladder while falling from it. There was no neurovascular compromise. Α CT scan of the right ankle with 3D reconstruction revealed a posteromedial fracture of the talar dome extending to and incorporating the medial tubercle of the posterior talar process, sparing the subtalar joint. (Figure 1A) The fracture was accessed through a posteromedial approach. The fracture fragment was found displaced posteriorly and distally in a rotated position. It was reduced and fixed with a 3.5 mm cancellous screw, which was inserted obliquely through the noncartilaginous medial talar wall under fluoroscopic control. (Figure 1B) The postoperative course was uncomplicated, and the patient was mobilized non–weight bearing with the operated leg in a back slab for 6 weeks. At the latest follow‐up at 3 years postoperatively, the fracture had united in an anatomical position and the patient was free of symptoms, with a painless, nearly full range of motion. The American Orthopaedic Foot and Ankle Score was 97/100. More specifically, the patient scored 40/40 in pain, 47/50 in function, and 10/10 in alignment. The MRI scan and radiographs at the latest follow‐up revealed a completely healed fracture with an intact articular surface. (Figure 1C,D).

Fracture of the talar dome including the tibiotalar joint is easily missed out in the clinical practice, so the Orthopaedic surgeon should be suspicious for the joint involvement in talar fractures in order to achieve a successful surgical management of the case.

## CONFLICT OF INTEREST

The authors declare they have no conflict of interest.

## AUTHOR CONTRIBUTIONS

E.P and C.K wrote the manuscript. D.E supervised the manuscript submission. M.M, K.Z, and G.K collected the data for the manuscript submission.

## ETHICAL APPROVAL

The case above was approved by the institutions’ ethical committee, and the patient included gave his informed consent prior to their inclusion in the case report.

## References

[1] Watanabe H , Majima T , Takahashi K , Kawaji H , Takai S . Split fracture of the posteromedial tubercle of the talus: case report and proposed classification system. J Foot Ankle Surg. 2016;56(1):1‐4. 10.1053/j.jfas.2016.02.001 26947002

[2] Albert P , Patel J , Katz JI , Loria F , Parnell J , Brenner M . Magnetic resonance imaging, computed tomography, and radiographic correlation of nonunion of the posteromedial tubercle of the talus: a case report. J Foot Ankle Surg. 2014;53:787‐790. 25179454 10.1053/j.jfas.2014.07.003

